# Pathergy of a Medial Heel Schwannoma

**DOI:** 10.7759/cureus.29463

**Published:** 2022-09-22

**Authors:** Abigail Durbin, Jack B Newcomer, Chase L Wilson

**Affiliations:** 1 Dermatology, University of Kentucky College of Medicine, Lexington, USA; 2 Medicine, University of Kentucky College of Medicine, Lexington, USA; 3 Dermatology, Elkhorn Dermatology, Georgetown, USA

**Keywords:** soft tissue tumors, oncodermatology, clinical dermatology, schwannoma, pathergy

## Abstract

Schwannomas are benign soft-tissue tumors derived from Schwann cells of the peripheral nervous system. They typically appear on the head, neck, or trunk, and are often asymptomatic or present with mild tenderness to palpation and numbness due to nerve compression. A 17-year-old male was referred to our dermatology clinic for evaluation and treatment of an asymptomatic, pink, flesh-colored subcutaneous nodule on the medial right heel. A biopsy was performed to rule out malignancy, with the pathology report consistent with the diagnosis of schwannoma. Following the biopsy, the patient developed a persistent, non-healing red-violaceous ulcerative plaque at the biopsy site, which persisted following additional electrodessication and silver nitrate application. Repeat biopsy showed persistent schwannoma and notably the absence of a pyogenic granuloma. The persistent ulceration following the initial biopsy is consistent with the phenomenon known as pathergy, which refers to exaggerated tissue reactivity in response to trauma. The patient eventually required surgical excision and a keystone flap for definitive treatment of the lesion. Although rare, we have demonstrated that pathergy can occur during surgical procedures on suspected schwannomas. Physicians should be aware of this possible complication so that they can provide anticipatory guidance for patients undergoing surgical procedures on undiagnosed cutaneous neoplasms for which a schwannoma is in the differential diagnosis.

## Introduction

A schwannoma is a benign soft-tissue tumor derived from Schwann cells in the peripheral nervous system [1]. These tumors typically present on the head, neck, or trunk but have also rarely been noted on the foot and other areas [2,3]. They are usually asymptomatic and slow-growing and have been identified across all ages. The gold-standard treatment of these tumors is surgical excision, but some tumors can be clinically observed if they are asymptomatic. In this report, we present a case of a schwannoma on the right heel of a 17-year-old male who underwent a punch biopsy to rule out malignancy and subsequently developed pathergy with persistent ulceration at the biopsy site. Ultimately, surgical excision was required to promote healing on the site of pathergy and from persistent ulceration.

## Case presentation

A 17-year-old male presented to the Elkhorn Dermatology clinic with a two-year history of an asymptomatic nodule located on his medial right heel. The patient had never sought a medical evaluation or undergone treatment for this lesion. On physical examination, a 2.2-cm pink, non-pulsatile, flesh-colored subcutaneous nodule was noted on the medial right heel. No distinctive morphological features were appreciated, which gave us a broad differential diagnosis including benign and malignant neural tumors, sweat duct tumors, and vascular tumors. A punch biopsy was performed and the pathology report revealed a diagnosis of schwannoma.

**Figure 1:**
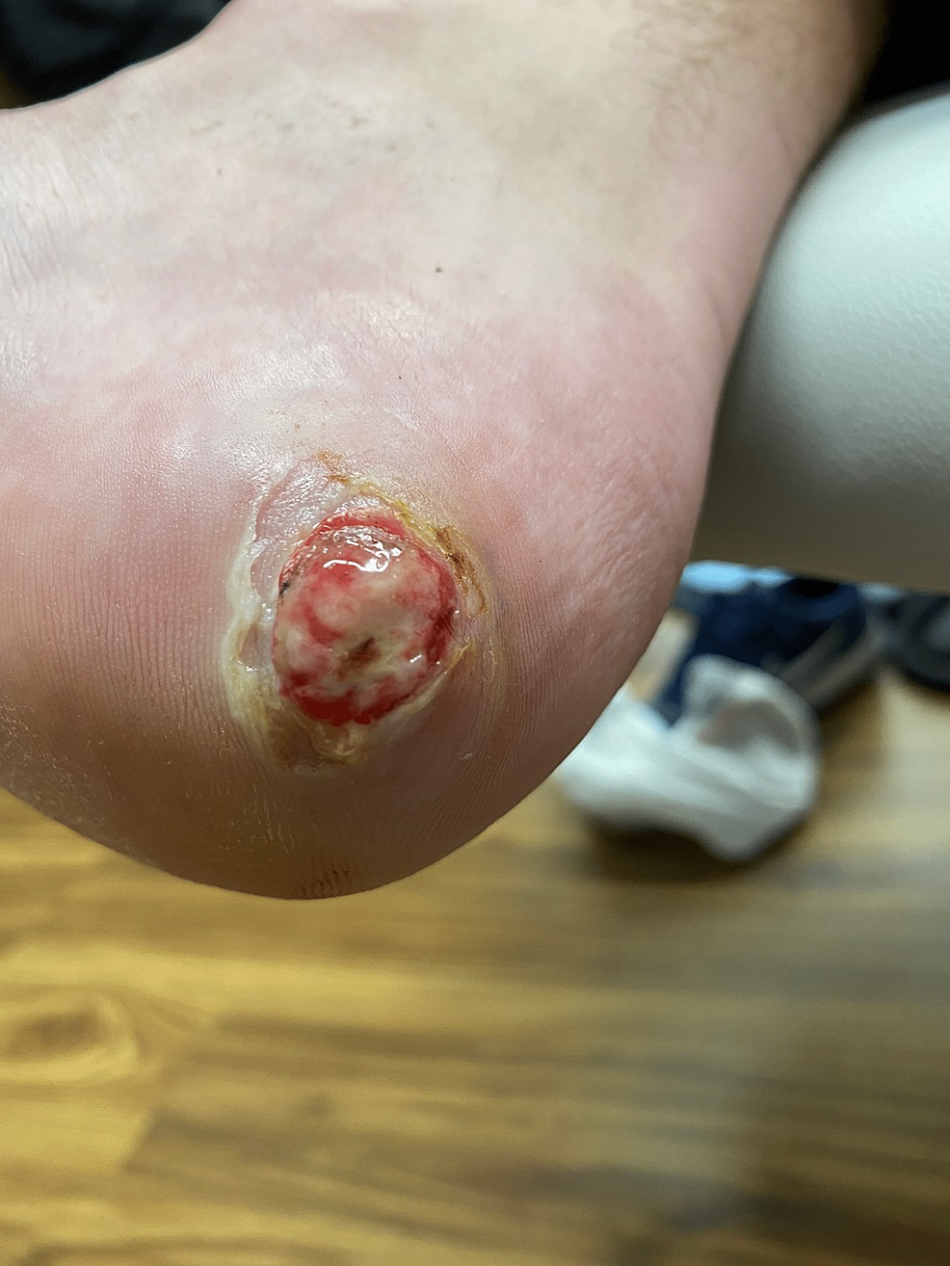
A new red-violaceous vascular plaque with ulceration present at the previous biopsy site

Three months after the punch biopsy, the patient returned to the clinic, stating that the biopsy site was not healing. He had developed a red-violaceous vascular plaque with ulceration at the biopsy site (Figure 1). The differential diagnosis for this plaque included pyogenic granuloma, exuberant granulation tissue, or persistent schwannoma. Shave removal with electrodessication of the plaque was performed, and pathology was again consistent with pre-existing schwannoma. Two weeks later, the site was still tender and intermittently bleeding; clinically, this was thought to be consistent with pathergy following the punch biopsy. Chemical cauterization was performed with topical silver nitrate to promote wound healing.

**Figure 2:**
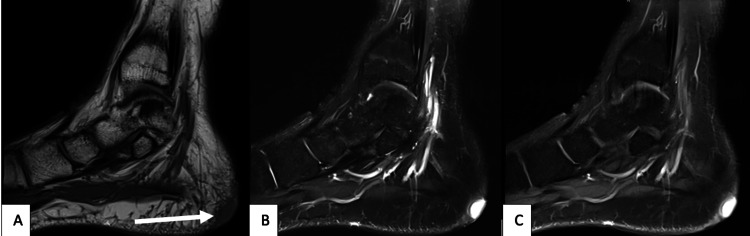
MRI of the patient Sagittal non-contrast T1 image (a) of the medial aspect of the foot showing low signal skin lesion in the posterior heel region (arrow); T2 image with fat suppression (b) showing bright signal lesion in the skin of the posterior foot, with the post-contrast image (c) redemonstrating intense and relatively homogenous enhancement of the lesion MRI: magnetic resonance imaging

The patient did not return to our clinic following the silver nitrate application. He was referred to plastic surgery at the University of Kentucky for evaluation and consideration of surgical excision given the failure of conservative treatment options. An MRI was performed, which showed a T1 hypointense, T2 hyperintense superficial intensely-enhancing focus within the subcutaneous fat along the medial heel, measuring approximately 17 x 9 x 15 mm (Figures 2A-2C). The surrounding anatomical structures in the foot appeared normal, which aligned with his initial asymptomatic presentation clinically. He ultimately underwent surgical excision and keystone flap approximately five months after initial evaluation at our dermatology clinic due to persistent ulceration and bleeding from the site. The patient was counseled to wear an air-cast boot for over one month to offload pressure from the wound site and allow the area to heal. Over the course of the next four months, the lesion healed without complication and exhibited no further signs of secondary change or hypersensitivity. The patient now has a well-healed scar at the incision site and is able to bear weight without symptoms.

## Discussion

The differential diagnosis for soft-tissue tumors of the foot and ankle is quite broad and includes a wide array of tumors of vascular, neural, connective tissue, and ductal origin [4]. In a retrospective study of 39,179 soft tissue tumors, Kransdorf et al. showed that only 8% of these tumors localized to the foot and ankle [5]. Among soft-tissue tumors localized to the foot and ankle, Hao et al. showed that only seven of 174 tumors (4%) were found to be schwannomas in a three-year period [1]. Both of these studies highlight the rarity of soft tissue tumors localizing to the foot, particularly schwannomas. Our case did not display any distinctive morphological features suggestive of one particular diagnosis; thus, a punch biopsy was performed to characterize the lesion and rule out malignancy.

Clinically, schwannomas mostly present with pain upon palpation and numbness due to nerve compression when symptomatic. Pathologic and radiographic analyses can both point toward diagnosis. On pathology, they are well-circumscribed and encapsulated tumors that often have two distinct histological regions, with a component of hypercellular spindle cells (Antoni A tissue), sometimes palisading around eosinophilic areas (Verocay bodies), as well as a hypocellular component (Antoni B tissue) in a background of loose connective tissue [6]. On MRI, schwannomas tend to appear similar in signal intensity to skeletal muscle on T1-weighted images and show high signal intensity on T2-weighted images [7]. Our patient underwent an MRI, which showed a T1 hypointense, T2 hyperintense, and intensely enhancing focus within the subcutaneous fat along the medial heel, measuring approximately 17 x 9 x 15 mm. The surrounding anatomical structures in the foot appeared normal, which aligned with the clinically asymptomatic presentation. MRI is thus a useful tool to determine the extent of tumor invasion and assist with surgical planning prior to resection.

Fewer than 15 cases of schwannoma recurrence after surgical excision have been reported in the literature. While our patient recovered from the operation without complications, his initial workup at our dermatology clinic was complex. His biopsy site from where we performed a punch biopsy had an exaggerated injury response, or pathergy [8]. Pathergy is characterized by a state of altered tissue reactivity in response to trauma and is often used as a diagnostic test of Behçet's syndrome with a needle prick to look for evidence of this phenomenon [9]. Pathergy can also be seen in other inflammatory conditions such as Crohn's disease, pyoderma gangrenosum, and Sweet's syndrome [10]. After the shave excision of the patient’s persistent schwannoma, the site became markedly ulcerated and developed protuberant beefy red granulation tissue, once again felt to be consistent with pathergy. To our knowledge, there have been no cases reported in the literature where a patient with a schwannoma has experienced pathergy following a biopsy procedure for tissue diagnosis. We believe the pathogenesis of pathergy in our patient could be related to disturbance of the localized tissue while performing a biopsy. Physicians should be aware of these sequelae so that they may properly educate their patients to anticipate them as possible negative outcomes secondary to biopsy, and subsequently recognize and treat the complication.

## Conclusions

Schwannomas are benign peripheral nerve tumors that are derived from Schwann cells. They are typically asymptomatic and do not require treatment but can be surgically removed for cosmetic purposes or if they are causing discomfort. Our patient underwent a biopsy of a nodular lesion on his medial heel to rule out malignancy, and although the pathology report was consistent with a benign schwannoma, he later developed a violaceous plaque with secondary ulceration overlying the tumor at the biopsy site. The persistent ulceration following the initial biopsy is consistent with the phenomenon known as pathergy, which refers to exaggerated tissue reactivity in response to trauma. Although rare, we have demonstrated that pathergy can occur following surgical procedures for suspected schwannomas. Physicians should be aware of the phenomenon of pathergy so that they can properly counsel their patients and provide anticipatory guidance for patients undergoing surgical procedures for undiagnosed cutaneous neoplasms for which a schwannoma is in the differential diagnosis.
